# Macrophage Cytokines Enhance Cell Proliferation of Normal Prostate Epithelial Cells through Activation of ERK and Akt

**DOI:** 10.1038/s41598-018-26143-8

**Published:** 2018-05-16

**Authors:** Tu Dang, Geou-Yarh Liou

**Affiliations:** 1 Center for Cancer Research and Therapeutic Development, Clark Atlanta University, Atlanta, GA USA; 2 Department of Biological Sciences, Clark Atlanta University, Atlanta, GA USA

## Abstract

Macrophage infiltrations (inflammation) are associated with prostate disorders such as prostatitis, prostatic hyperplasia and prostate cancer. All prostate disorders have elevated cell proliferation, and are initiated from normal prostate epithelial cells. To date, the mechanism of how macrophages regulate normal prostate epithelial cell proliferation remains largely unknown. Using a 3D co-culture system, we here show that Raw 264.7 macrophages increased cell proliferation of normal prostate epithelial PZ-HPV-7 cells. In addition, these Raw 264.7 macrophages expressed higher levels of Ym1 and CD206. We further identify macrophage-secreted cytokines including CCL3, IL-1ra, osteopontin, M-CSF1 and GDNF as mediators for potentiating PZ-HPV-7 cell proliferation in 3D. All these cytokines differentially activated ERK and Akt. Blockade of both kinases through their inhibitors hindered macrophage-induced cell proliferation of PZ-HPV-7 cells. Hence, our data provide mechanistic insight of how inflammation may contribute to development of prostatic diseases at a very early stage through augment of cell proliferation of normal prostate epithelial cells.

## Introduction

Prostate disorders including prostatitis, prostatic hyperplasia, and prostate cancer are the most commonly encountered health issue among men older than 50 years. The etiology and pathogenesis of the prostate disorders, so far, remains unclear. All these disorders are associated with an increase of inflammation^1–6^ and elevated cell proliferation of prostate cells^7–12^. A sustained inflammatory cell environment and uncontrolled cell proliferation, both of which can lead to tumorigenesis. In despite of a large body of evidence that inflammation promotes cancer initiation and development in many types of cancers^13,14^, how inflammation positively contributes to prostate disorders, particularly prostate cancer, is still an ongoing debate. This is because of certain conflicting results from clinical studies^15–20^.

In response to infections of bacteria or pathogens, and injury, immune cells are rapidly activated to defend the body from further damage, known as inflammation. During inflammation, macrophages are the major type of immune cells activated to execute their tasks including pathogen killing and wound healing^21,22^. In addition, genetic mutations, epigenetic alterations, age, obesity and environmental stimuli such as diet have been demonstrated to generate a more inflammatory environment by upregulating reactive oxygen species (ROS)^23–28^. Depending on their activators, macrophages are classified into either classically-activated/M1 or alternatively-activated/M2 subtypes. M1 macrophages activated by lipopolysaccharide and interferon γ destroy pathogens through producing nitric oxide and inflammatory cytokines^29,30^. Meanwhile, M2 macrophages activated by interleukin 4, interleukin 13 and other can repair wounds, synthesize extracellular matrix and promote cell growth through their secreted anti-inflammatory cytokines^31,32^.

We, herein, show that macrophage-secreted cytokines are mediators to increase cell proliferation of normal prostate epithelial cells in a 3D cell culture system. Moreover, these macrophage cytokines activate ERK and Akt, and inhibition of both protein kinases abolish macrophage-medicated cell proliferation. Therefore, we provide evidence for mechanistic insight into how inflammation leads to a set-up for initiating prostate diseases through induction of a higher cell proliferation rate of normal prostate epithelial cells.

## Results

### Macrophages promote cell proliferation of normal prostate epithelial cells

**Figure 1:**
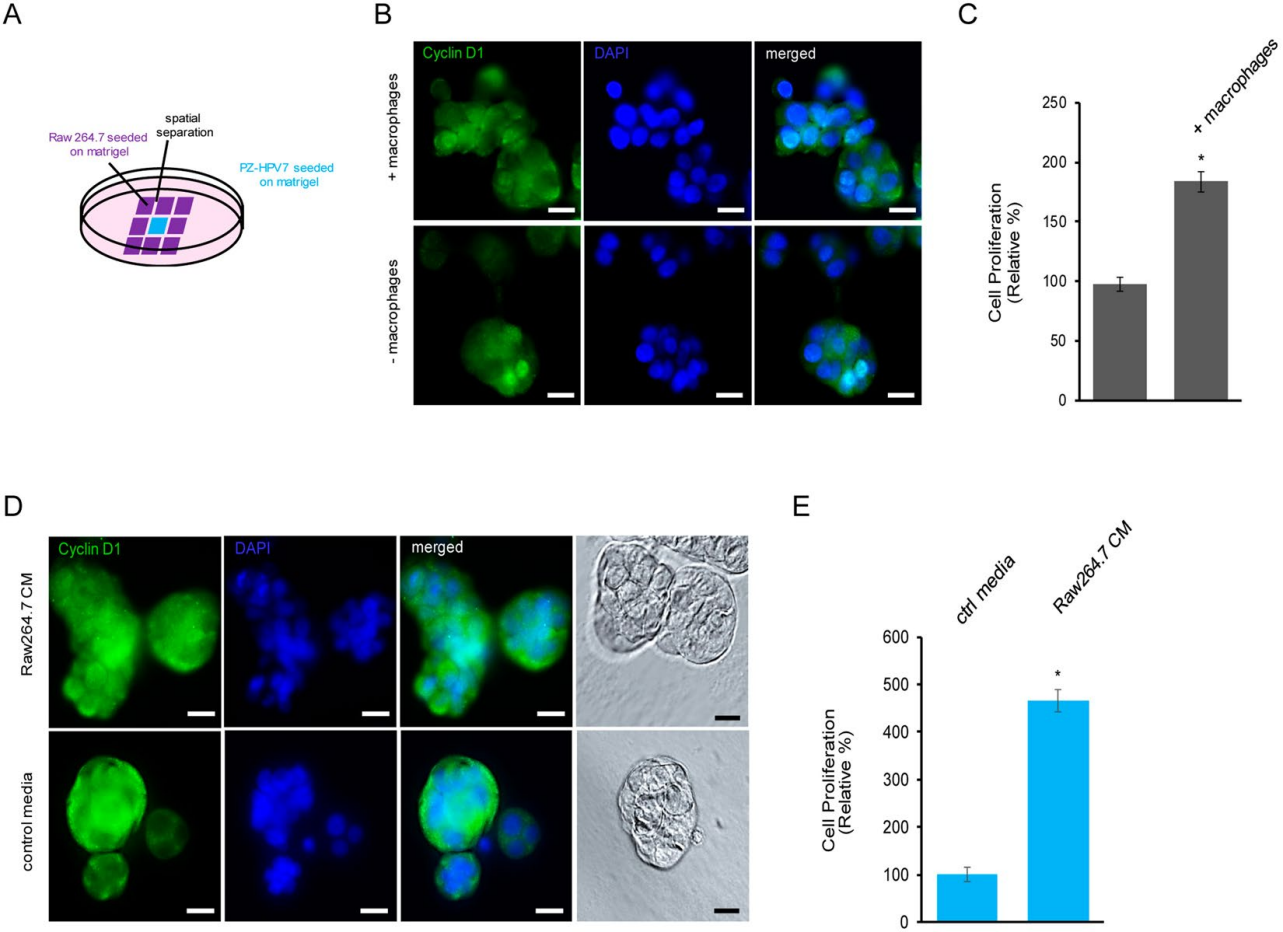
Macrophages promote cell proliferation of normal prostate epithelial cells. (**A**) A diagram for demonstrating cell co-cultivation of normal prostate epithelial PZ-HPV-7 cells (blue area) and Raw 264.7 cells (purple area). Cells were plated on matrigel in µ-Slides (Ibidi) and overlaid with KSF media. (**B**) PZ-HPV-7 cells grown on matrigel in 3D with or without Raw264.7 cells in a co-culture system (described in A) were fixed and immuno-stained with cyclin D1 (green). DAPI (blue) was used to visualize all cell nuclei. Scale bar: 20 µm. (**C**) Cell proliferation index of PZ-HPV-7 cells that were cultured with or without Raw 264.7 cells in 3D was quantified. *p < 0.05. (**D**) PZ-HPV-7 cells were cultured on matrigel in 3D in Raw 264.7-conditioned media or control media. Cells were fixed and immune-stained with cyclin D1 (green). DAPI (blue) was used to visualize all cell nuclei. Scale bar: 20 µm. (**E**) Cell proliferation index of PZ-HPV-7 cells that were cultured in 3D with control media or Raw 264.7-conditioned media was quantified. *p < 0.05.

To decipher the effect of macrophage-mediated process on cell proliferation of normal prostate epithelial cells, we co-cultured Raw 264.7 macrophages with immortalized normal prostate PZ-HPV-7 epithelial cells on matrigel in a three dimensional setting. These two types of cells were seeded in separated compartments of a co-cultivation system (see scheme in Fig. 1A), which only allows cells to share soluble substances released in the media instead of physical contacts. As reported previously^33^, PZ-HPV-7 cells when cultured in 3D formed acinar clusters (Fig. 1B). In order to directly evaluate the cell proliferation under a 3D environment without any extra artificial inputs, we fixed cells in 3D and then used nuclear cyclin D1 as a readout for cell proliferation^34–36^ by immunostaining cells with a cyclin D1 specific antibody. As shown in Fig. 1B, when co-cultured with Raw 264.7 macrophages in 3D, more PZ-HPV-7 cells expressed nuclear cyclin D1. Results from quantification of PZ-HPV-7 cell clusters demonstrated a statistically significant increase of cell proliferation of PZ-HPV-7 cells in the presence of Raw 264.7 macrophages (Fig. 1C). Given that a physical interaction is not required for inducing PZ-HPV-7 cell proliferation by macrophages, we next used Raw 264.7-conditioned media to treat PZ-HPV-7 cells. As shown in Fig. 1D,E, Raw 264.7-conditioned media increased numbers of nuclear cyclin D1 positive cells of PZ-HPV-7. Moreover, Raw 264.7-conditioned media had a better effect on PZ-HPV-7 cell proliferation as compared to co-cultivation of Raw 264.7 macrophages. Altogether, these data indicated a promoting role of macrophages in proliferation of normal prostate epithelial cells.

### Macrophage-secreted CCL3 and IL-1ra mediate cell proliferation of normal prostate epithelial cells

**Figure 2:**
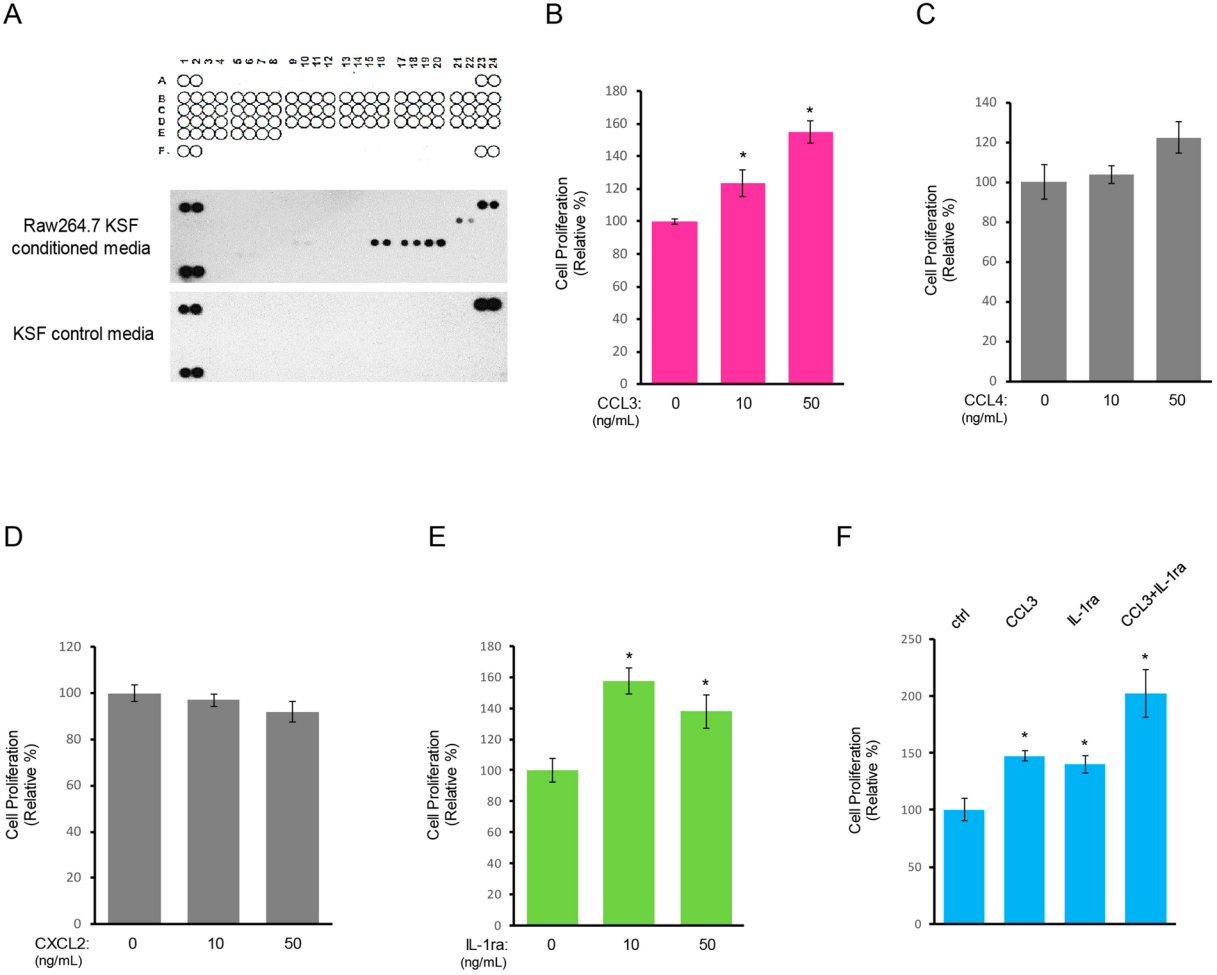
CCL3 and IL-1ra secreted by macrophages mediate cell proliferation of normal prostate epithelial cells. (**A**) Raw 264.7-conditioned media and control media were subjected to a mouse cytokine array for identifying the cytokines present in Raw 264.7-conditioned media. A1/2, A23/24, and F1/2 contain positive controls, and F23/F24 contains negative controls. (**B–E**) PZ-HPV-7 cells grown on matrigel in 3D were treated with the cytokines identified from Raw 264.7-conditioned media including CCL3 (B), CCL4 (C), CXCL2 (**D**) and IL-1ra (**E**) at the indicated concentrations. Cell proliferation index of PZ-HPV-7 cells under these treatments was quantified. *p < 0.05. (F) PZ-HPV-7 cells grown on matrigel in 3D were treated control/ddH_2_O, CCL3 (50 ng/mL), IL-1ra (10 ng/mL) or both for 48 h. Cell proliferation index under these conditions was quantified. *p < 0.05.

To identify the macrophage-secreted factors that promoted PZ-HPV-7 cell proliferation in 3D matrigel culture, we performed cytokine profiler arrays for analyzing Raw 264.7-conditioned media. Among 40 tested cytokines (Fig. S1), only four cytokines including CCL3, CCL4, CXCL2 and IL-1ra were present in Raw 264.7-conditioned media (Fig. 2A). We next tested the capability of these identified cytokines on PZ-HPV-7 cell proliferation in 3D matrigel culture. Upon CCL3 stimulation, cell proliferation of PZ-HPV-7, as judged by the cyclin D1 nuclear localization, was increased in a dose dependent manner (Fig. 2B). Similarly, IL-1ra was also capable of inducing proliferation of PZ-HPV-7 cells (Fig. 2E). Neither CCL4 nor CXCL2 affected cell proliferation of normal prostate PZ-HPV-7 epithelial cells in a 3D culture setting (Fig. 2C and D). A dual treatment of CCL3 and IL-1ra in PZ-HPV-7 cells additionally elevated cell proliferation as compared to either CCL3 or IL-1ra alone (Fig. 2F). This suggests that these two cytokines may activate different downstream signaling pathways that led to cell proliferation.

### Osteopontin, M-CSF1 and GDNF upregulated by Ym1^+^/CD206^+^ macrophages promote cell proliferation of normal prostate epithelial cells

**Figure 3:**
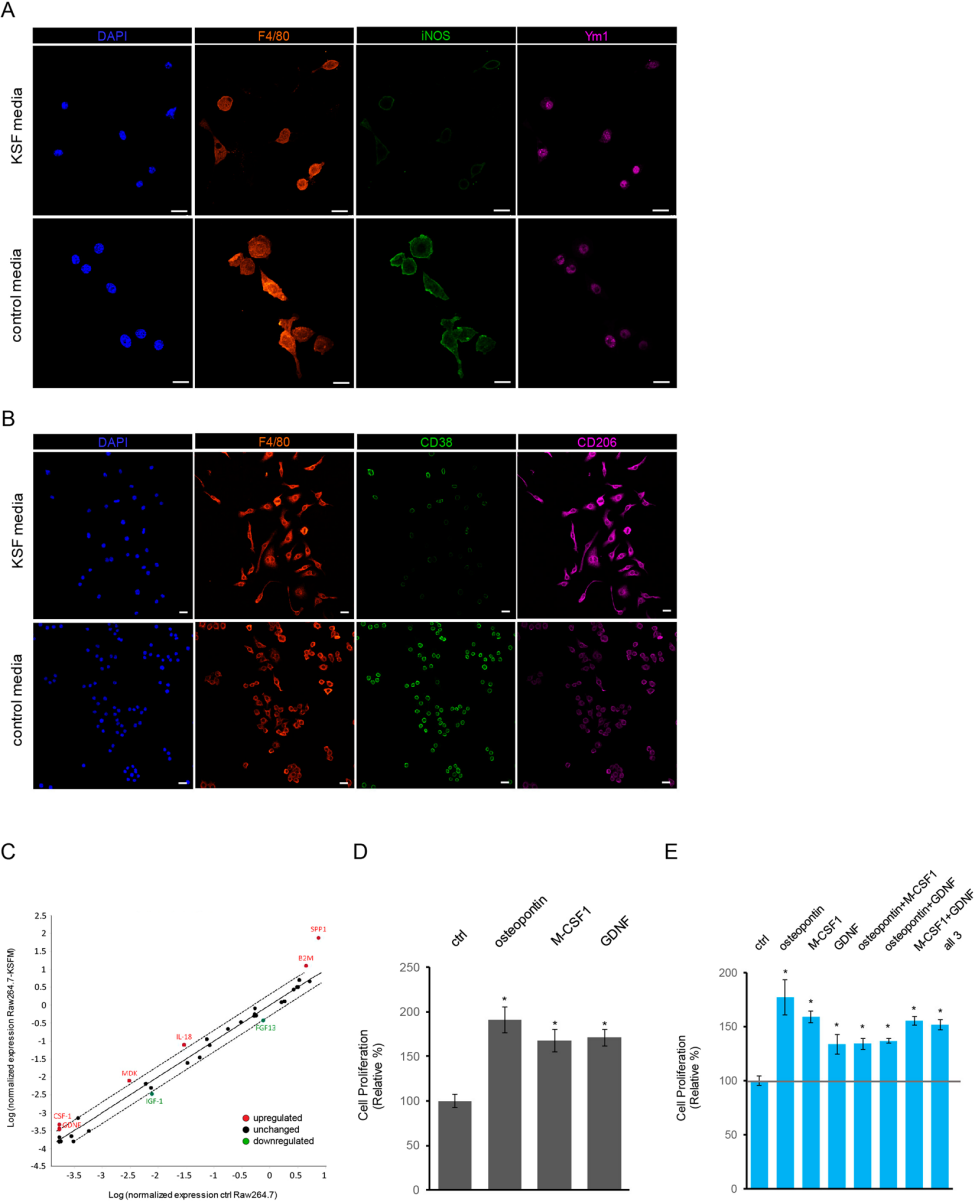
Osteopontin, M-CSF1 and GDNF upregulated by Ym1^+^/CD206^+^ macrophages promote cell proliferation of normal prostate epithelial cells. (A) Raw 264.7 cells were cultured in control media or KSF complete media for 24 h. Cells were immuno-stained with F4/80 (orange, pan-macrophage marker), iNOS (green, M1 macrophage marker) and Ym1 (pink, M2 macrophage marker). Cell nuclei were visualized by DAPI staining. Scale bar: 20 µm. (**B**) Similar to A, Raw 264.7 cells were cultured in control media or KSF complete media and immuno-stained with F4/80 (orange, pan-macrophage marker), CD38 (green, M1 macrophage marker) and CD206 (pink, M2 macrophage marker). Cell nuclei were visualized by DAPI staining. Scale bar: 20 µm. (**C**) RNA samples from Raw 264.7 cells cultured in control media or KSF complete media were subjected to growth factor PCR arrays for identifying the growth factors upregulated in Raw264.7 macrophages grown in KSF complete media. A scatter plot was shown for upregulated growth factors (red), unaltered growth factors (black) and downregulated growth factors (green). (**D**,**E**) PZ-HPV-7 cells grown on matrigel in 3D were treated either control/ddH_2_O, osteopontin (250 ng/mL), M-CSF1 (100 ng/mL), GDNF (50 ng/mL) or combination of these cytokines as indicated. Cell proliferation index under these conditions was quantified. *p < 0.05 as compared to the control/ddH_2_O.

In response to stimulations under different cell environments, macrophages are either M1 pro-inflammatory or M2 immune-suppressive for executing their distinct tasks, e.g. killing the infected cells by foreign pathogens for M1 macrophages vs repairing wounds for M2 macrophages^21,22^. To investigate which subtype of macrophages was present in our 3D matrigel co-culture system that resulted in PZ-HPV-7 cell proliferation, we immune-stained the macrophages cultured in either control media or co-culture media with several well-established markers including iNOS, CD38 for M1 macrophages^29,37^, and Ym1, CD206 for M2 macrophages^30,31^ respectively (Fig. 3A,B). Indeed, all Raw 264.7 macrophages expressed F4/80, a pan-macrophage marker (Fig. 3A,B). In addition, higher levels of Ym1 and lower levels of iNOS were expressed in Raw 264.7 macrophages that generated Raw 264.7-conditioned media to promote PZ-HPV-7 cell proliferation in 3D matrigel culture. Consistent result was observed using CD38 as alternative M1 macrophage marker, and CD206 as additional M2 macrophage marker (Fig. 3B), suggesting that the macrophages resulted in PZ-HPV-7 cell proliferation were Ym1^+^/CD206^+^ M2 subtype.

To screen more potential cytokines that were upregulated in these macrophages and actually functioned as growth factors to modulate cell proliferation of PZ-HPV-7, we utilized real time qPCR arrays. Among 84 examined genes expressed in the macrophages cultured in the control or co-culture media, 6 genes were upregulated at least 2-fold higher and 2 genes were downregulated at least 2-fold lower in the macrophages grown in the co-culture media than these in the control media. (Fig. 3C). In addition, 76 genes remained at similar levels (within a 2-fold difference). The 6-upregulated genes included Spp1 (known as osteopontin), M-CSF-1, GDNF, B2M, IL-18 and MDK; and the 2-downregulated genes were IGF-1 and FGF13. Out of the 8 genes that showed different expressions in macrophages, Spp1, M-CSF1 and GDNF held the strongest change. Therefore, we next focused on effects of Spp1, M-CSF1 and GDNF on PZ-HPV-7 cell proliferation in a 3D matrigel culture setting. As shown in Fig. 3D, additions of either recombinant osteopontin, M-CSF1 or GDNF significantly increased cell proliferation of PZ-HPV-7 cells as judged by the percentage of cells expressing nuclear cyclin D1. However, combined treatments of these three cytokines did not result in any synergistic growth as compared to each single treatment (Fig. 3E), suggesting that osteopontin, M-CSF1 and GDNF may activate similar signaling pathways to mediate cell proliferation.

### Macrophages potentiate cell proliferation of normal prostate epithelial cells through activation of ERK and Akt

**Figure 4:**
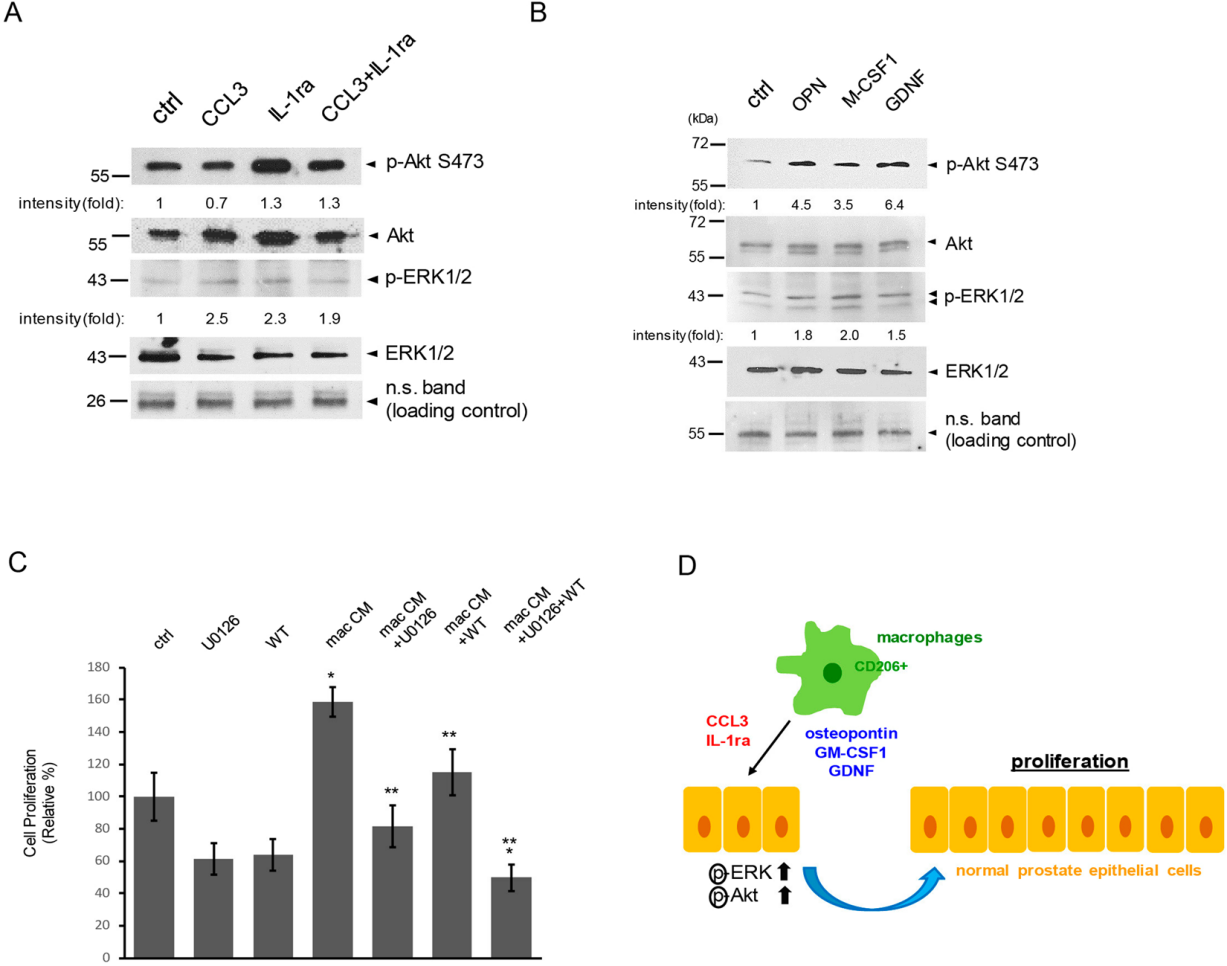
Macrophages potentiate cell proliferation of normal prostate epithelial cells through activation of ERK and Akt. (**A**) PZ-HPV-7 cells grown on matrigel in 3D were treated with CCL3, IL-1ra or both for 48 h. Cell lysates were collected and subjected to immunoblotting for examining the protein of interests as indicated. (**B**) Similar to A, PZ-HPV-7 cells cultured on matrigel in 3D were treated with control/ddH_2_O, osteopontin (OPN), M-CSF1 or GDNF for 48 h. Cell lysates were collected from 3D culture and subjected to immunoblotting for examining the protein of interests as indicated. (**C**) U0126 or wortmannin (WT) was added to 3D cultures of PZ-HPV-7 cells in the presence or absence of Raw 264.7-conditioned media for 48 h. Vehicle (DMSO) was used as control. Cell proliferation index under these conditions was quantified. *p < 0.05 as compared to the control/vehicle. **p < 0.05 as compared to Raw 264.7-conditioned media. (**D**) A scheme to summarize how macrophages may promote cell proliferation of normal prostate epithelial cells.

In response to growth factor and cytokine stimulations, activation of mitogen-activated protein kinase (MAPK, also known as ERK) and Akt leads to cell proliferation, differentiation and survival under physiological conditions^38–40^. To test the activation status of these two protein kinases in our 3D matrigel culture of PZ-HPV-7 cells that are stimulated with the identified macrophage-regulated cytokines as described previously, we subjected cell lysates collected from the 3D culture system to immunoblots analysis. Addition of cytokines CCL3, IL-1ra or both to PZ-HPV-7 cells for 1 h activated ERK, but not Akt (Fig. S2A). Treatment of IL-1ra for 48 h slightly activated Akt signaling in PZ-HPV-7 cells (Fig. 4A). In addition, either CCL3 or IL-1ra treatment for 48 h upregulated ERK activation. Treatment of either osteopontin, M-CSF1 or GDNF in the PZ-HPV-7 cells that are grown on matrigel in a 3D setting for 1 h slightly increased phosphorylated Akt and ERK (Fig. S2B). 48 h treatment of either these cytokines strongly elevated both Akt and ERK activation (Fig. 4B). Notably, a higher-fold increase in levels of the phosphorylated Akt than that of the phosphorylated ERK was detected. These results indicated a differential activation between Akt and ERK in response to various macrophage-regulated cytokines. To examine if ERK and Akt activated by macrophage-secreted factors indeed mediate cell proliferation, we used inhibitors U0126 and Wortmannin to target ERK and Akt respectively in our 3D culture of PZ-HPV-7 cells in the presence or absence of Raw 264.7 macrophage-conditioned media. Although a disruption of big clusters of PZ-HPV-7 cells formed in 3D cultures was observed when either inhibitor was added, it had minimal (not statistically significant) effects on cell proliferation (Fig. S3, and Fig. 4C). Treatment of U0126 or Wortmannin decreased cell proliferation that was induced by Raw 264.7-conditioned media (Fig. 4C). Moreover, a dual inhibition of both kinases had a synergistic effect and further lowered the cell proliferation rate of PZ-HPV-7 cells.

Altogether, these data suggested that when co-cultured with normal prostate epithelial cells in a 3D environment, macrophages were polarized to Ym1^+^/CD206^+^ subtype. These Ym1^+^/CD206^+^ macrophages promoted cell proliferation of normal prostate epithelial cells through their secreted cytokines, including CCL3, IL-1ra, osteopontin, M-CSF1 and GDNF, which activate ERK and Akt signaling (Fig. 4D).

## Discussion

Increased cell proliferation is one of the major characteristics of prostate disorders including benign prostatic hyperplasia (BPH) and prostate cancer. These disorders typically progress with age advancement especially in senior males. Other risk factors for these prostatic disorders include obesity and African ancestry. All these described contributing factors are associated with elevated inflammation^41–45^. Macrophages are the major type of immune cells activated during inflammation. How macrophages affect normal prostate epithelial cells to initiate prostate diseases remains majorly unclear.

Our results indicate that macrophage-upregulated cytokines including CCL3, IL-1ra, osteopontin, M-CSF1 and GDNF are mediators for inducing cell proliferation of normal prostate epithelial PZ-HPV-7 cells in 3D cultures (Figs 2B,E and 3D,E). It is the first time to link these cytokines to cell proliferation of the immortalized and normal prostate epithelial cells. In pathological conditions, these cytokines have been reported to regulate cell proliferation, migration, invasion and metastasis^46–54^. In prostate disease BPH, macrophage-secreted CCL3 was downstream target of androgen receptor (AR), which is also secreted by macrophages, to promote prostate stromal cell proliferation^6^. In a mouse model of experimental autoimmune prostatitis, CCL3 was shown to induce chronic pelvic pain^55^. IL-1ra from CD11b^+^Gr1^+^ myeloid cells has been demonstrated to overcome cell senescence of PTEN null prostate tumors in mice^56^. Transplanting IL-1ra knockout myeloid cells to the PTEN-null mice accelerated cell senescence. In CD133^+^ stem/progenitor cells of prostate cancer and CD133^+^ C4–2 prostate cancer cells, IL-1ra expressed by CD133^+^ cells mediates testicular nuclear receptor 4 (TR4)-induced drug resistance^57^.

In addition to CCL3 and IL-1ra, we also showed that macrophage-upregulated cytokines osteopontin, M-CSF1 and GDNF in the co-culture media were capable of inducing cell proliferation of normal prostate epithelial cells (Fig. 3D,E). Notably, among these identified cytokines, osteopontin has been reported to promote cell proliferation and cell survival in several types of cells including smooth muscle cells, quiescent prostate epithelial cells, erythroblasts and neural stem cells^58–61^. It also regulates cell adhesion, migration and bone remodeling, which associate with bone metastasis in breast cancer and prostate cancer^62,63^. In prostate cancer, higher expressions of osteopontin were detected in the patient tissue samples of prostate cancer as compared to these of normal and BPH^64^. Osteopontin also elevates the anchorage-independent growth, intravasation potential, and EGF-dependent cell proliferation in cultured human prostate cancer cell lines^65–67^. Our data demonstrated that the macrophages that upregulated the cytokines to increase cell proliferation of the immortalized normal prostate epithelial cells in the co-culture system were similar to alternatively activated/M2 macrophages (Fig. 3A,B), which express Ym1 and CD206^30,31^. This result is in support of the notion that M2-like macrophages participate in cell proliferation and tissue repair^21,68,69^.

Our data showed that CCL3 and IL-1ra activated ERK rather than Akt kinase in the normal prostate epithelial cell line (Fig. 4A). Interesting, cytokines osteopontin, M-CSF1 and GDNF that were also upregulated by macrophages in the 3D culture system strongly activated Akt than ERK (Fig. 4B). This suggests that macrophages ensure to activate both ERK and Akt signaling through secreting various cytokines. When stimulating PZ-HPV-7 cells with CCL3 or IL-1ra for 1 h, increased phospho-ERK at similar levels to that of 48 h treatment was observed (Fig. 4A and Fig. S2A). It suggested a direct activation of ERK pathway by CCL3 and IL-1ra. Activation of Akt and ERK by other macrophage cytokines including osteopontin, M-CSF1 and GDNF may be indirect since both protein kinases required a longer time to gain much higher activities (Fig. 4B and Fig. S2B). When treating cells with Akt or ERK inhibitor, each inhibitor did not significantly reduce the basal level cell proliferation in a 3D environment (Fig. 4C, first 3 lanes). Notably, a disruption of PZ-HPV-7 cell cluster formation was observed (Fig. S3). However, these smaller cell clusters of PZ-HPV-7 were viable and proliferative. Altogether, these results indicated that the used dose of U0126 and wortmannin was not toxic to cells, and that both kinases may participate in cell-cell and/or cell-matrix interactions.

In summary, we provided evidence to demonstrate that macrophages potentiate cell proliferation of immortalized normal prostate epithelial cells in a 3D environment. We also delineated the mechanism of how macrophages achieve this task by activation of ERK and Akt kinases through macrophage-secreted cytokines including CCL3, IL-1ra, osteopontin, M-CSF1 and GDNF. Given that these immortalized normal prostate epithelial cells have increased cell proliferation in response to inflammatory/macrophage stimulations and that elevated cell proliferation is reported in certain prostatic diseases, our findings may provide plausible explanations on how inflammation contributes to giving rise on these prostatic diseases.

## Materials and Methods

### Cell lines, antibodies and reagents

Raw 264.7 macrophage cells (ATCC) were cultured in RPMI 1640 containing 10% FBS and 100 U/ml penicillin/streptomycin. PZ-HPV-7 cell (ATCC) were cultured in Keratinocyte Serum Free media containing 0.05 mg/mL bovine pituitary extract and 5 ng/mL human recombinant EGF. Cells were maintained in a 37 °C incubator supplemented with 5% CO_2_. To obtain Raw 264.7-conditioned media, 2.5 × 10^5^ cells per 6-well dish were grown in Keratinocyte Serum Free complete media, and supernatant was collected. Conditioned media were freshly prepared for each experiment. Both anti-F4/80 and anti-cyclin D1 antibodies were from Thermo Fisher Scientific; anti-iNOS and anti-CD206 antibodies were obtained from Abcam. The anti-CD38 antibody was from Novus Biologicals; anti-Akt, anti-phospho-Akt (ser473) and anti-ERK antibodies were purchased from Cell Signal Technology. The anti-phospho-ERK1/2 antibody was from Santa Cruz Biotechnology, and the anti-Ym1 antibody was obtained from STEMCELL Technologies. Human recombinant CCL3, CCL4, CXCL2, IL-1ra, M-CSF1 and GDNF were purchased from PeproTech. Human recombinant osteopontin was obtained from BioLegend. Matrigel was from Corning. U0126 and wortmannin (WT) were obtained from Cell Signaling Technology. Other reagents used are described in the specific experiment sections.

### 3D culture of PZ-HPV-7 cells and Treatment

PZ-HPV-7 single cells were plated on top of 50% matrigel and stimulated with the indicated recombinant proteins with the indicated concentrations or vehicle control. For U0126 or wortmannin (WT) treatment, 10 µM U0126 or 0.5 µM WT were added to the cells simultaneously with Raw 264.7-conditioned media or control media after cells were seeded on matrigel.

### Cell proliferation assay

PZ-HPV-7 cells grown in 3D matrigel culture with the indicated stimulation/treatment for 48 h were rinsed twice with PBS (140 mM NaCl, 2.7 mM KCl, 8 mM Na_2_HPO_4_ and 1.5 mM KH_2_PO_4_ [pH 7.2]), fixed with 4% paraformaldehyde-PBS for 20 min, permeabilized with 1% TritonX-100 in PBS for 10 min, 0.5% TritonX-100 in PBS for 20 min, 0.1% SDS in PBS for 1 min, and blocked in 10% goat serum 1 h at room temperature. Samples were incubated with anti-cyclin D1 antibody (1:250) overnight at 4 °C. Samples were washed three times with PBS containing 0.05% Tween-20. Secondary antibodies Alexa Fluor 488 goat-anti-rabbit (1:500) from Thermo Fisher Scientific were added for 30 min at room temperature. After another three washes of PBST, DAPI was used to label cell nuclei. Images were visualized in ibidi mounting media. Serial sections of images (Z-stack) were captured by a Zeiss Axiovert 200 M inverted fluorescent microscope with a 20x objective lens. Each 3D image were reconstructed from Z-stack images. Numbers of nuclear cyclin D1 were counted in randomly five 3D images per condition. Cell proliferation index was calculated using the ratio: numbers of nuclear cyclin D1/numbers of all cells (DAPI). For the experiment using U0126 and wortmannin, cell proliferation index was used solely based on DAPI counts due to the interference of these two compounds on the formation of cell clusters of PZ-HPV-7.

### Cellular lysates and Immunoblotting

Cells were washed twice with PBS, lysed in buffer A (50 mM Tris/HCl [pH 7.4], 1% TritonX-100, 150 mM NaCl, and 5 mM EDTA [pH 7.4]) supplemented with protease inhibitor cocktail (Thermo Fisher Scientific), incubated on ice for 30 min and centrifuged at 4 °C, 14000 rpm for 10 min. The supernatants were were subjected to SDS-PAGE, and resolved proteins were transferred onto nitrocellulose membranes. The membranes were blocked in 5% BSA in TBST (50 mM Tris.HCl [pH 7.6], 150 mM NaCl, 0.05% Tween 20) and incubated with the antibodies of interest in 5% BSA in TBST overnight at 4 °C. The appropriate horseradish peroxidase-conjugated 2° antibodies were applied for 30 min at room temperature. Samples were visualized with ECL and X-ray film.

### Immunocytochemistry and Confocal microscope imaging

Cells grown in the indicated conditions were rinsed twice with PBS, fixed in 4% paraformaldehyde-PBS for 20 min, permeabilized with 0.1% TritonX-100 in PBS for 10 min, and blocked in 10% goat serum 1 h at room temperature. Samples were incubated with primary antibodies (anti-F4/80 (1:200); anti-iNOS (1:250); anti-Ym1 (1:200); anti-CD38 (1:200); anti-CD206 (1:1000)) overnight at 4 °C. Samples were washed three times with PBS containing 0.05% Tween-20. Secondary antibodies (Alexa Fluor 488, 594 and 647 obtained from Thermo Fisher Scientific at 1:500) were added for 30 min at room temperature. Samples were washed three times with PBST. Images were captured using a LSM 700 confocal laser scanning microscope (Zeiss) with a 10x objective lens.

### Cytokine array

The cytokines secreted by Raw 264.7 macrophages were examined using the Proteome Profiler Mouse Cytokine Array Kit (R&D Systems) according to the manufacturer’s instructions.

### Growth factor PCR array

The growth factors expressed by Raw 264.7 macrophages were determined using the RT^2^ Profiler PCR Array PAMM-041Z (Qiagen) according to the manufacturer’s instructions. Genes of the examined growth factors changed greater than 2-fold were plotted in a scatter plot.

### Statistical analysis

Data are presented as means ± SE. P-values were acquired with the Student’s *t* test using Prism (GraphPad Software), and P < 0.05 is considered statistically significant.

## Electronic supplementary material

Supplemental Information
