# Supplementary material for: Macrophage Cytokines Enhance Cell Proliferation of Normal Prostate Epithelial Cells through Activation of ERK and Akt
Source: Sci Rep. 2018 May 16;8:7718. doi: 10.1038/s41598-018-26143-8 (PMC5955920; doi:10.1038/s41598-018-26143-8)

Supplementary Information for

# Macrophage Cytokines Enhance Cell Proliferation of Normal Prostate Epithelial Cells through Activation of ERK and Akt

Tu Dang[1], and Geou-Yarh Liou[1,2,3]

[1] Center for Cancer Research and Therapeutic Development, Clark Atlanta University, Atlanta, GA, USA

[2] Department of Biological Sciences, Clark Atlanta University, Atlanta, GA, USA

[3] Corresponding author: Geou-Yarh Liou, Clark Atlanta University, Room 4005D, Thomas Cole Research Building, 223 James P. Brawley Drive SW, Atlanta, GA 30314. Phone: (404) 880-6981, FAX: (404) 880-6756, E-mail: gliou@cau.edu

## Supplemental Figure S1

| Coordinate | Terget/control | Alternative Nomenclature | Raw 264.7 |
|---|---|---|---|
| A1,A2 | Postive Control | Control (+) | |
| A23, A24 | Postive Control | Control (+) | |
| B1, B2 | BLC | CXCL13/BCA-1 | |
| B3, B4 | C5a | Complement Component 5a | |
| B5, B6 | G-CSF | – | |
| B7, B8 | GM-CSF | – | |
| B9, B10 | I-309 | CCL1/TCA-3 | |
| B11, B12 | Eotaxin | CCL11 | |
| B13, B14 | sICAM-1 | CD54 | |
| B15, B16 | IFN-γ | – | |
| B17, B18 | IL-1α | IL-1F1 | |
| B19, B20 | IL-1β | IL-1F2 | |
| B21, B22 | IL-1ra | IL-1F3 | |
| B23, B24 | IL-2 | – | |
| C1, C2 | IL-3 | – | |
| C3, C4 | IL-4 | – | |
| C5, C6 | IL-5 | – | |
| C7, C8 | IL-6 | – | |
| C9, C10 | IL-7 | – | |
| C11, C12 | IL-10 | – | |
| C13, C14 | IL-13 | – | |
| C15, C16 | IL-12 p70 | – | |
| C17, C18 | IL-16 | – | |
| C19, C20 | IL-17 | – | |
| C21, C22 | IL-23 | – | |
| C23, C24 | IL-27 | – | |
| D1, D2 | IP-10 | CXCL10/CRG-2 | |
| D3, D4 | I-TAC | CXCL11 | |
| D5, D6 | KC | – | |
| D7, D8 | M-CSF | – | |
| D9, D10 | JE | CCL2/MCP-1 | |
| D11, D12 | MCP-5 | CCL12 | |
| D13, D14 | MIG | CXCL9 | |
| D15, D16 | MIP-1α | CCL3 | |
| D17, D18 | MIP-1β | CCL4 | |
| D19, D20 | MIP-2 | CXCL2 | |
| D21, D22 | RANTES | CCL5 | |
| D23, D24 | SDF-1 | CXCL12 | |
| E1, E2 | TARC | CCL17 | |
| E3, E4 | TIMP-1 | – | |
| E5, E6 | TNFα | TNFSF1A | |
| E7, E8 | TREM-1 | – | |
| F1, F2 | Postive Control | Control (+) | |
| F23, F24 | PBS (Negative Control) | Control (-) | |

**Supplemental Figure S1: Indentified cytokines in the Raw 264.7-conditioned media.** Table contains the cytokine position (left column) and identity (middle columns) from the mouse cytokine profiler array. The enriched cytokines present in the Raw 264.7-conditioned media are highlighted in blue (right column).

**Supplemental Figure S2**

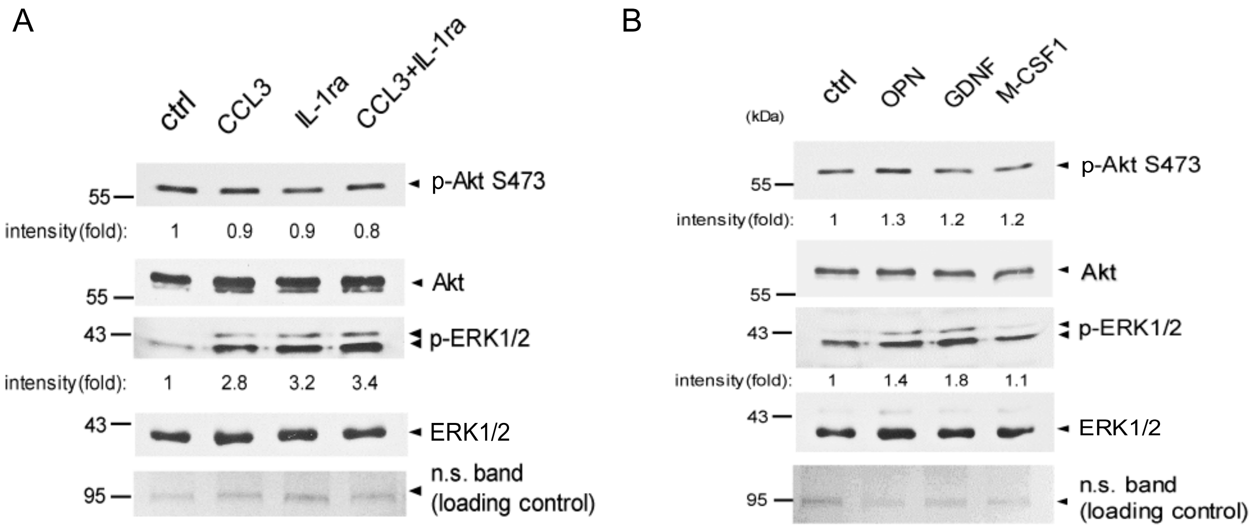


**Supplemental Figure S2: Status of activated Akt and activated ERK by a short stimulation of macrophage-induced cytokines.** (A) PZ-HPV-7 cells grown on matrigel in 3D were treated with CCL3, IL-1ra or both for 1 h. Cell lysates were collected and subjected to immunoblotting for examining the protein of interests as indicated. (B) Similar to A, PZ-HPV-7 cells cultured on matrigel in 3D were treated with control/ddH2O, osteopontin (OPN), M-CSF1 or GDNF for 1 h. Cell lysates were collected from 3D culture and subjected to immunoblotting for examining the protein of interests as indicated.

**Supplemental Figure S3**

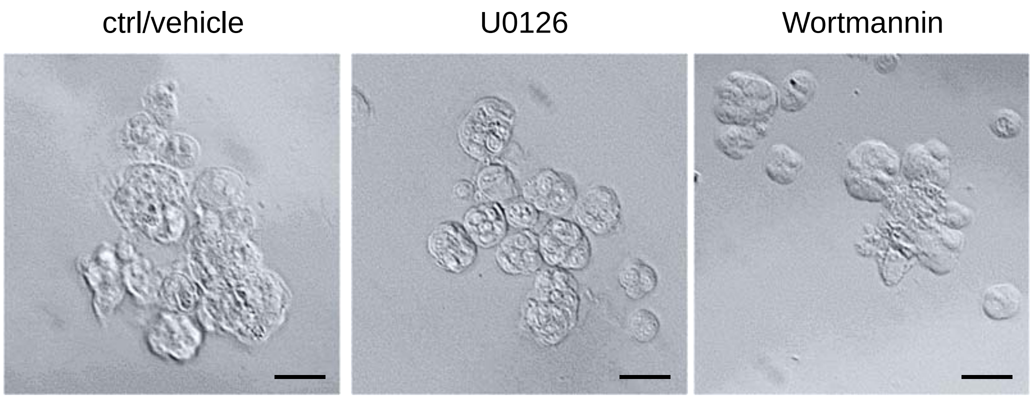


**Supplemental Figure S3: U0126 or Wortmannin treatment disrupted cell clusters of PZ-HPV-7 in 3D matrigel culture.** PZ-HPV-7 cells were treated with either control/vehicle (DMSO), U0126 or Wortmannin, and simultaneously seeded on top of matrigel in 3D for 2 days for evaluating cell proliferation. Bright field images were taken at the endpoint to document the size of the cell clusters under each condition. Scale bar: 50 µm.

**Supplemental Figure S4, uncropped immnoblots for Figure 4A and Figure 4B**

Figure 4A

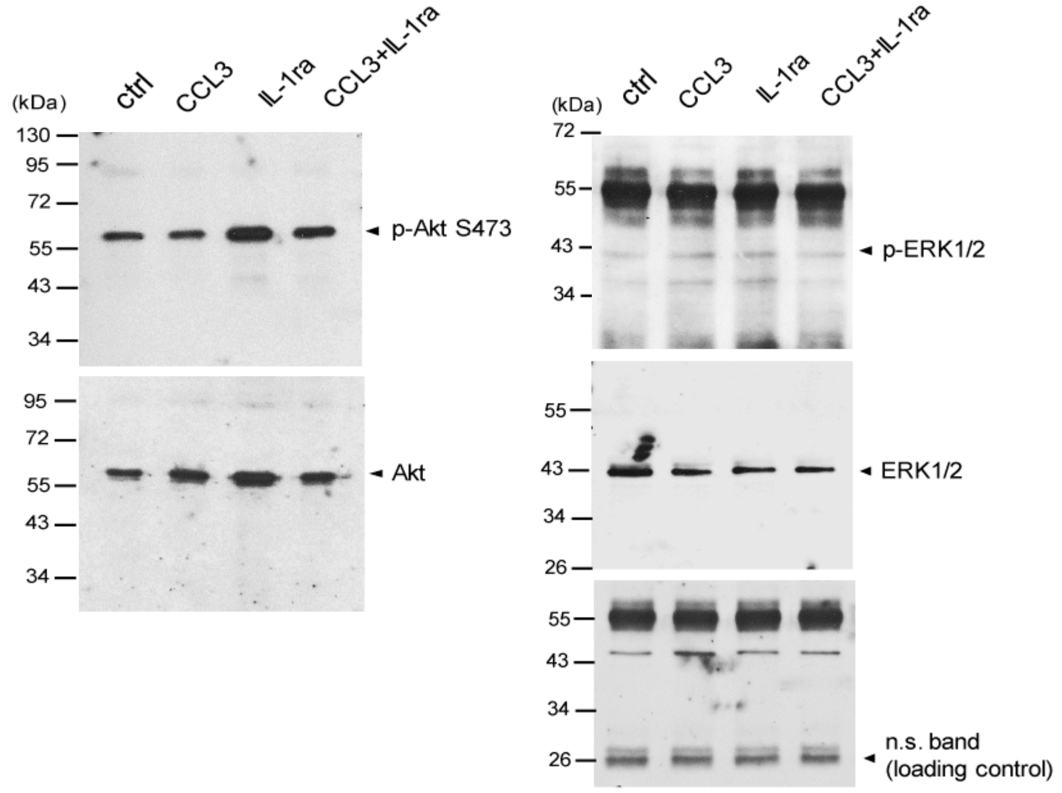


Figure 4B

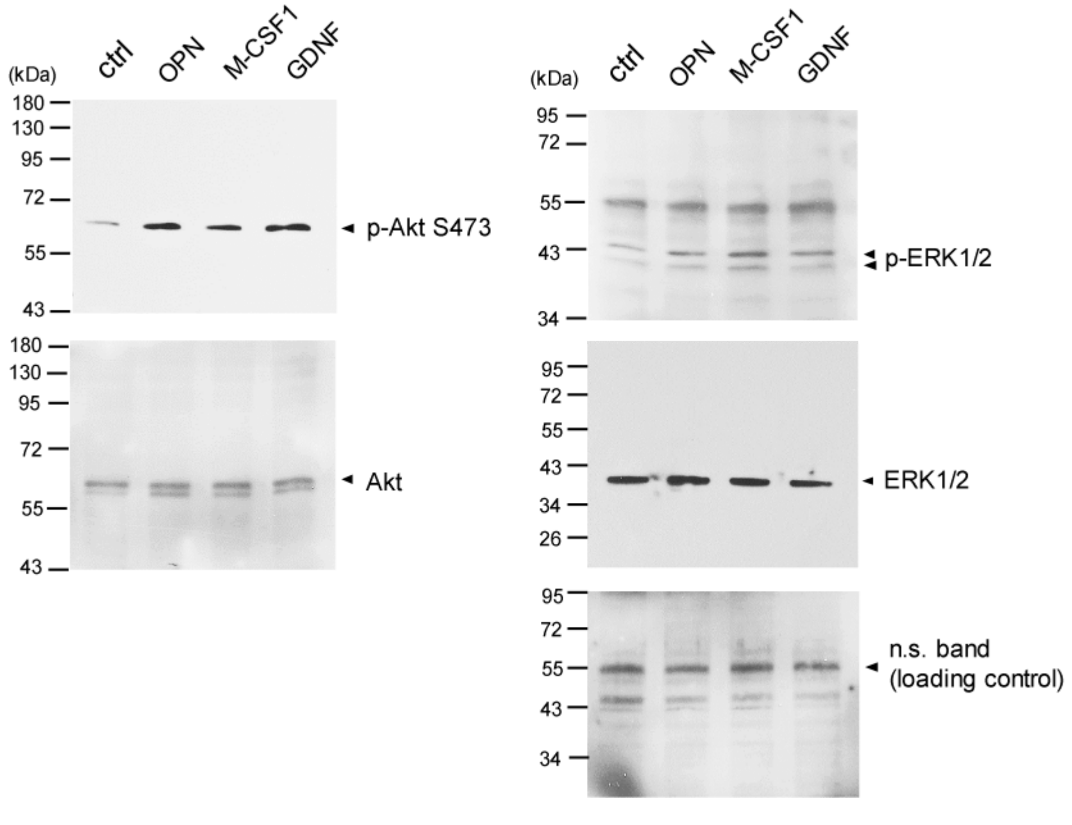

**Supplemental Figure S5, uncropped immnoblots for Supplemtal Figure 2A and Figure 2B**

Figure S2A

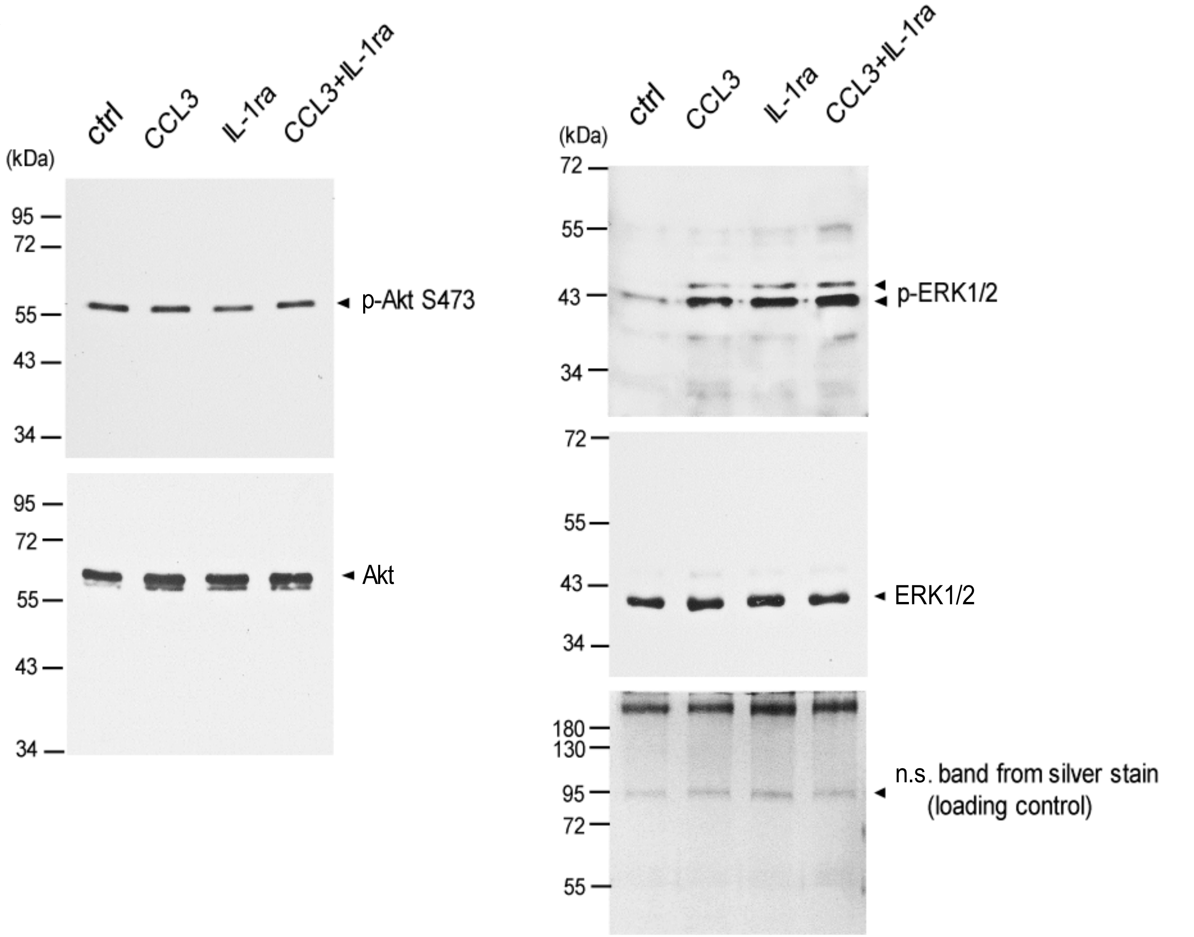


Figure S2B

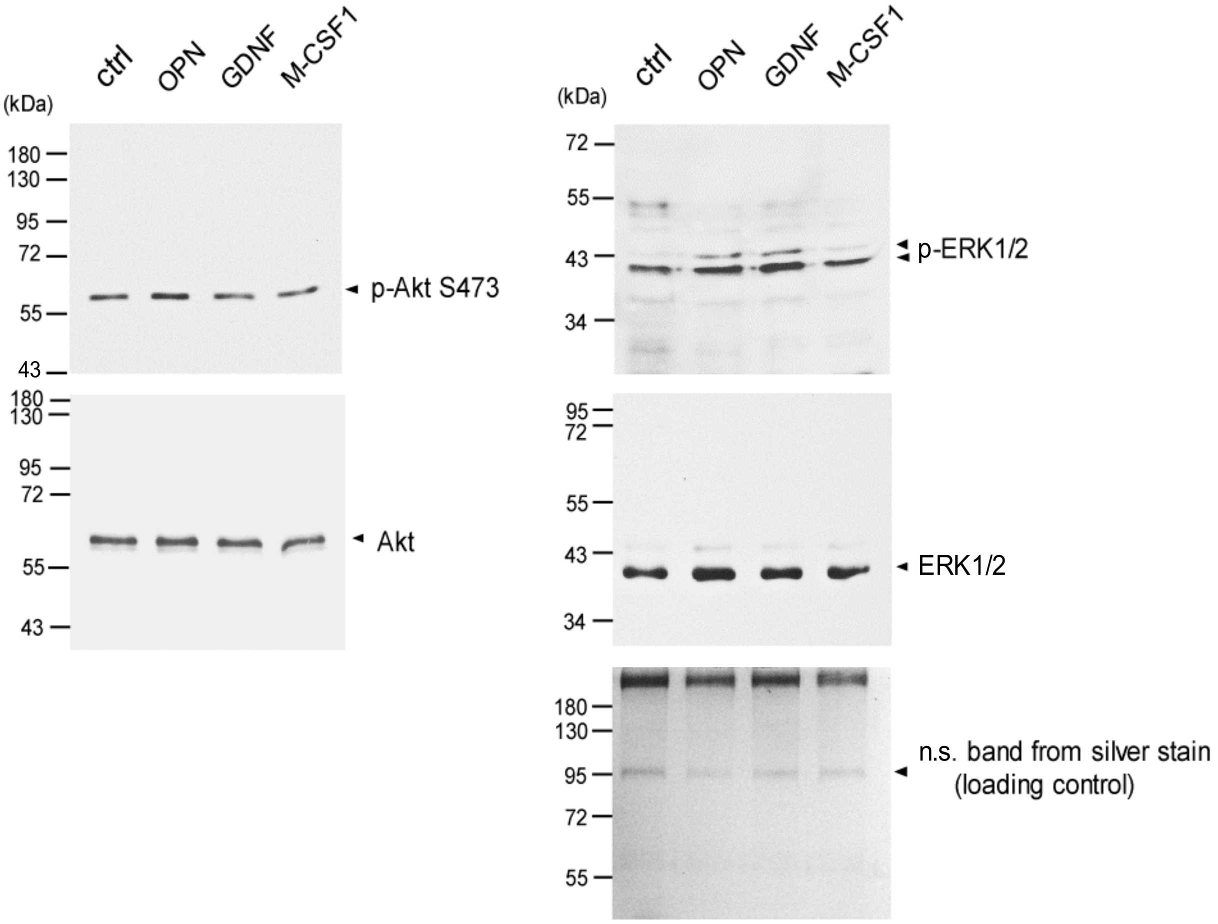

Supplement: Supplementary file 1 — Supplemental Information [file 41598_2018_26143_MOESM1_ESM.pdf]
